# Which Data Do Elementary School Teachers Use to Determine Reading Difficulties in Their Students?

**DOI:** 10.1177/0022219420981990

**Published:** 2021-01-15

**Authors:** Alexandra M. A. Schmitterer, Garvin Brod

**Affiliations:** 1 DIPF—Leibniz Institute for Research and Information in Education, Frankfurt am Main, Germany; 2 Goethe University Frankfurt am Main, Germany

**Keywords:** diagnostics, teachers, reading intervention

## Abstract

Small-group interventions allow for tailored instruction for students with learning difficulties. A crucial first step is the accurate identification of students who need such an intervention. This study investigated how teachers decide whether their students need a remedial reading intervention. To this end, 64 teachers of 697 third-grade students from Germany were asked to rate whether a reading intervention for their students was “not necessary,” “potentially necessary,” or “definitely necessary.” Independent experimenters tested the students’ reading and spelling abilities with standardized tests, and a subsample of 370 children participated in standardized tests of phonological awareness and vocabulary. Findings show that teachers’ decisions with regard to students’ needing a *reading* intervention overlapped more with results from standardized spelling assessments than from reading assessments. Hierarchical linear models indicated that students’ spelling abilities, along with phonological awareness and vocabulary, explained variance in teachers’ ratings over and above students’ reading skills. Teachers thus relied on proximal cues such as spelling skills to reach their decision. These findings are discussed in relation to clinical standards and educational contexts. Findings indicate that the teachers’ assignment of children to interventions might be underspecified, and starting points for specific teacher training programs are outlined.

Learning to read is one of the most important skills to acquire in early childhood and equips children with an important tool for lifelong learning (Cunningham & Stanovich, 1997; Esser et al., 2002; McBride, 2019). Thus, effective reading interventions should be provided to children who struggle with reading acquisition. One important key to an effective intervention is the accurate identification of children with problems in reading or reading-related abilities (e.g., Compton et al., 2012; Reynolds & Shaywitz, 2009; Snowling, 2013; Tunmer & Greaney, 2010).

Education systems rely on different selection methods for remedial intervention. For example, in the United States, some federal states assign teachers a “gating function,” and a third party such as a therapist identifies children who need a more detailed learning assessment. In other educational settings, teachers themselves use information from standardized and formalized assessments to track students’ individual development and assign them to separate groups with different learning strategies or goals (for the Response-to-Intervention approach, see Compton et al., 2006). In many educational settings (e.g., in European countries and regions), teachers whose diagnostic approaches are unformalized decide which children need an intervention (Ingram et al., 2004; Schildkamp & Kuiper, 2010). Little is known about the information teachers with unformalized approaches use to reach their decisions. This study addresses this gap in research by exploring how teachers reach their decisions about whether their students need a reading intervention, and how these decisions relate to estimations based on standardized assessments.

Previous studies have indicated that an accurate identification of struggling readers is a necessary condition for an effective intervention (e.g., Compton et al., 2006, 2012; Connor et al., 2013; Förster et al., 2018; Förster & Souvignier, 2015). These studies were based on standardized identification processes established to combine standardized summative and formative assessments with specific types of interventions (i.e., Response-to-Intervention approaches). It is yet difficult to translate results from the studies and apply them to educational environments without a formalized identification of children with reading problems (Ingram et al., 2004; Schildkamp & Kuiper, 2010). Thus, in those educational contexts, information on how teachers reach their decisions about their students’ achievement levels is important to studying the aptness of applied reading intervention programs.

To the authors’ knowledge, no extensive studies have addressed how teachers reach their decisions about who to identify for a reading intervention. However, a number of studies have focused on teachers’ assumptions about their students’ academic achievements by comparing them with standardized assessments. For example, Martin and Shapiro (2011) asked teachers to predict the scores students would achieve on a standardized reading test. Results showed a moderate correlation of teachers’ estimations and actual test scores, with teachers overestimating the reading ability of their students. A moderate agreement between teachers’ ratings of their students’ reading or spelling abilities and standardized test scores was also found (see Eckert et al., 2006; Feinberg & Shapiro, 2003, 2009; Hoge & Coladarci, 1989; Martin & Shapiro, 2011; Schabmann & Schmidt, 2009). In line with these findings, Begeny and colleagues (2008) found that teachers’ ratings showed a strong overlap with standardized oral reading fluency assessment for children who performed well but not for children with lower achievement levels.

However, contrary findings have also been reported. In two studies, preschool teachers rated children’s literacy skills on several subcomponents of reading (i.e., letter-sound knowledge, letter-name knowledge). Taken together, these ratings led to the conclusion that teachers were rather good at identifying children at risk for literacy difficulties (Taylor et al., 2000; Titley et al., 2014). Thus, studies focusing on a range of skills that teachers might teach and observe in their students found more positive results. However, these studies were conducted with kindergarten teachers instead of elementary school teachers and based on questionnaires that guided the identification process rather than measuring the teachers’ own decision. Such a scenario is usually not available to teachers in educational environments in which identification assessments are not formalized.

Overall, this body of research indicates that teachers typically do not assess students’ reading abilities in the same way as standardized assessments that focus on specific abilities. On the contrary, when more skills are considered and the identification process is guided, teachers seem to be able to reliably identify at-risk children. The teachers’ decision making should thus be based on a variety of skills. Moreover, experimental settings are difficult to compare with a less formalized environment. Most of the studies were conducted in an educational environment with rather established standardized assessments (summative or formative assessments), that is, several regions of the United States. Evidence is still missing for teachers’ decision-making processes who do not rely on standardized assessments and are not closely guided in their decision making. The aim of this study was to identify skills that those teachers use to reach their decisions about which children need a reading intervention. To do so, we reviewed literature on skills that indicate reading difficulties and the literature that more generally focuses on how teachers use various sources of information for instructional decision making.

## Skills Indicating Reading Difficulties

According to clinical standards which are relatively clearly defined, the prevalence of individuals with a reading or spelling disorder varies between 3% and 11% (i.e., Moll et al., 2014; Pennington et al., 2012; Snowling, 2013) depending on the diagnostic criteria that are adhered to (Fischbach et al., 2013; McBride, 2019; Snowling & Hulme, 2012; Wyschkon et al., 2009). Legal standards of clinical diagnoses refer to expert classifications (i.e., *Diagnostic and Statistical Manual of Mental Disorders* [5th ed.; *DSM-5*]: American Psychiatric Association [APA], 2013; International Statistical Classification of Diseases and Related Health Problems German Modification [ICD-10-GM]: World Health Organization [WHO], 2020) according to which reading, spelling, and mathematics disorders are treated as subcategories of one diagnosis (i.e., Specific Learning Disorders). Furthermore, reading and spelling disorders are indicated if reading (i.e., reading accuracy, reading fluency, or reading comprehension) and spelling skills are substantially below age or class level in standardized assessments and not otherwise explained by low intelligence, inadequate schooling, visual or auditory impairments, or lack of language proficiency. However, distinctions of subcategories differ between classification systems.

The WHO (2020) classifies spelling disorders both combined with reading disorders and separately. Specifically, the WHO points out that dyslexia is often used as a term for combined reading and spelling difficulties. The APA (2013) treats reading and spelling disorders as separate disorders that often appear together. The lack of more distinct subcategories within reading and between reading and spelling has been criticized (e.g., Snowling & Hulme, 2012). Specifically in shallower orthographies (e.g., German), isolated forms of reading and spelling disorders have been identified (Moll & Landerl, 2009). Furthermore, difficulties in reading accuracy or reading fluency have been found to be related to different causes (e.g., phonological processing deficits) other than isolated difficulties with reading comprehension (e.g., language comprehension deficits). Thus, children with isolated reading accuracy or fluency difficulties (i.e., dyslexia) require different interventions than children with spelling difficulties (i.e., dysgraphia) or reading comprehension difficulties (i.e., poor comprehension) (Gough & Tunmer, 1986; McBride, 2019; Snowling & Hulme, 2012). Therefore, there is some dispute with regard to the definition of diagnoses of reading disorders in the scientifc and clinical communities.

In summary, clinical definitions of reading disorders are complex and to some extent vague with regard to their dissociation of different reading and spelling disorders (Castles et al., 2018; Schatschneider & Torgesen, 2004). Based on these definitions, a variety of skills indicate reading and reading-related disorders, including basic and advanced reading abilities, spelling abilities (either as validating or confining indicator), phonological processing abilities, and language comprehension abilities (e.g., vocabulary knowledge). To the best of our knowledge, no study has so far explored which of these skills teachers rely on and how strongly they rely on each skill in their decision about their students’ need of a reading intervention.

## Teachers’ Use of Data in General Instructional Decisions

Teachers in many educational environments are free to choose which information or methodology they use to identify children with reading disorders. It is, therefore, important to understand which information teachers use for instructional decisions (Schildkamp & Kuiper, 2010). Lai and Schildkamp (2013) distinguished input data (i.e., student background), process data (i.e., classroom instructions), context data (i.e., instructional guidelines), and outcome data (i.e., student assessments; see also Booher-Jennings, 2005; Ikemoto & Marsh, 2007). Moreover, teachers have been found to base their decisions more on experience and intuition than on systematically collected data (Ingram et al., 2004). Their decisions might hence be vulnerable to perceptive and cognitive biases. For example, some evidence suggests that even if systematic data are provided to teachers (i.e., formative assessments), they choose data that confirm prior beliefs (confirmation bias) or directly come from their own daily practices (attainable cues; Dhami et al., 2004; Gelderblom et al., 2016; O’Reilly et al., 1989; Tversky & Kahnemann, 1974). Thus, literature suggests that teachers might not only refer to children’s different skills to identify reading difficulties but also more generally different types of information (i.e., outcome data, context data). Furthermore, teachers’ interpretation of data might be obscured by cognitive biases. Thus, in this study, we also aimed to collect some information on what kind of data teachers used as sources of information and considered cognitive biases as a source of explanation for teachers’ decisions.

## This Study

The overarching aim of this exploratory study was to find indicators that predict teachers’ identifications of children in need of remedial reading intervention. To this end, we evaluated the overlap of teachers’ unformalized decisions about their students’ need for additional reading intervention with categorizations by standardized tests assessing a number of reading-related skills. Moreover, we aimed to identify types of data (i.e., outcome data, input data) teachers report using to reach their decision and which of these data teachers relied on most. Finally, classroom-level effects were explored to test whether teachers’ decisions were influenced by the average performance levels within their classroom.

Data from a German study with 64 elementary school teachers and 697 third-grade students were analyzed. Teachers were asked to rate whether their students needed an additional reading intervention on a short rating scale matching pragmatic decisions in schools. Results of the ratings were compared with diagnostic categorization of standardized tests for reading on the word, sentence, and text level as well as for spelling. Moreover, teachers were asked to indicate what information they relied on. Furthermore, multilevel analyses were run to identify skills of children associated with teachers’ decision making and to identify possible biases. Based on previous literature (e.g., Martin & Shapiro, 2011), decisions and the results of ratings based on standardized reading and spelling assessments of students were expected to show a moderately strong agreement. Furthermore, we explored which literacy skills would be associated with teachers’ decisions. With regard to cognitive biases, we expected teachers’ decisions to be related to the average achievement level in their classroom as opposed to an absolute criterion of reading difficulties. Ultimately, we aimed to identify indicators that are associated with, and perhaps bias, teachers’ unformalized decision making about their students’ need of a reading intervention.

## Method

### Participants

Data presented here were collected as part of a large-scale study of third-grade students in the regions of Hessia and Lower Saxony in Germany. Across two school years, data from 942 students from 77 classes in 35 elementary schools were collected. Participants were recruited with the help of the Ministry of Education in the state of Hessia, which distributed information about the study to teachers in the state, as well as through recruitment at the University of Hildesheim. Ultimately, 77 teachers participated in the study, 64 of whom provided ratings for their students’ eligibility for additional reading intervention. Therefore, the data set used for this study consisted of 64 classes with 803 students in 32 schools. Both teachers and the students’ parents consented to participation prior to the study.

#### Teachers

The 64 teachers were predominantly female (*N*_male_ = 3) and their mean age was 40.60 years (*SD* = 8.79). Additional information about the sample was collected with a questionnaire, which was completed by 53 teachers. According to their responses, teachers had on average 13.49 years (*SD* = 6.79, range = 4–30) of teaching experience in general and 5.61 years (*SD* = 4.85, range = 0–20) of teaching experience with third graders. With regard to their university training, 45 out of 53 teachers were trained for elementary school teaching and two were originally or additionally trained in special education. The remaining teachers were originally trained for a different school type or in a different profession.

#### Students

Of 803 participating students, 749 completed group test sessions in which reading, spelling, and nonverbal intelligence tasks were administered by trained student research assistants. The other students were either absent during the test days (e.g., due to illness) or had missing data. An additional 52 children were excluded from the analysis because they had a nonverbal IQ score (Grundintelligenztest Skala 1 [Basic Intellgience Test Scale 1] [CFT1-R]; Weiß & Osterland, 2013) of below 70 and, therefore, would fall under separate criteria for special needs education (i.e., global learning difficulties, mental disabilities).

Of the remaining 697 children, an average of 10.89 students per class (*SD* = 4.14) participated in the study. In addition, teachers nominated 370 (53%) students to participate in individual sessions. In these sessions, tasks on reading-related verbal abilities and phonological working memory were administered by trained student research assistants to collect more information about students’ reading-related language skills. Due to limited time for assessment in the respective schools, teachers could name up to eight children for individual sessions. They were asked to prioritize children with reading difficulties and then add children based on their own criteria for representativeness of the class. On average, 5.78 children (*SD* = 2.81) of each class participated in individual sessions.

Additional background information about the children participating in the group and individual sessions is presented in Table 1. This background information was obtained through parent and student questionnaires, which overall indicated that participants came from a diverse socioeconomic background. Furthermore, distributions of gender, age, and socioeconomic background were similar in the samples of the group and individual sessions. The number of children whose parents indicated a literacy impairment was slightly higher in the individual sessions, which was expected because teachers had been asked to first indicate children with reading problems for individual sessions. Finally, the percentage of children learning German as a second language was higher in the sample of children participating in individual sessions.

**Table 1.** Descriptive Statistics of Background Information About Students.

	Group sessions		Individual sessions	
Baseline Characteristics	*n*	%	*n*	%
Gender				
Female	386	55	199	54
Male	311	45	171	46
Language background				
Monolingual German	414	60	163	44
Bilingual with German	106	15	49	13
German as a second language	177	25	158	43
Impairments and support				
Literacy impairments^a,b^	68	11	57	18
Received support^a,c^	42	61	9	16
Other impairments^a,d^	119	19	69	22
Received support^a,c^	45	38	21	30
	*M*	*SD*	*M*	*SD*
Age in years; months	8;9	5.81	8;10	6.28
Socioeconomic background^e^	54.28	16.9	53.08	17.19

a Based on the information given by 91% of the parents. ^b^Isolated or nonisolated literacy impairments. ^c^For example, additional lessons in school, speech or psychotherapy. ^d^For example, speech impairments or isolated dyscalculia. ^e^Operationalized by HISEI (Highest International Socio-Economic Index of Occupational Status), Ganzeboom et al. (1992) and Ganzeboom (2010), range in group sessions = 15–89, range in individual sessions = 17–89.

#### Educational environment

In Germany, elementary education is based on a predefined curriculum with annual or semiannual learning goals that are defined at the level of federal states. On average, about 27% of the time in the German elementary curriculum is dedicated to reading, writing, and literature (Organisation for Economic Co-operation and Development [OECD], 2019). Within this structure, schools and teachers decide about the progression, methods, and material. In Hessia and Lower Saxony (Hessisches Kultusministerium [HKM], 2017b; Niedersächsisches Kultusministerium [MK], 2005), teachers and schools are responsible for identifying children with specific learning problems and for creating individual curricula with additional learning opportunities (e.g., additional lessons, individualized material, oral exams). The identification of children with specific learning needs is, however, not based on regulated procedures. Hessia provides a guideline with material and information about learning disorders and ways to diagnose them (HKM, 2017b). According to this guideline, spelling disorders can occur separately from reading disorders. Furthermore, the guideline refers to standardized and unstandardized instruments for diagnostic purposes. Teachers also learn about clinical standards of diagnosis as part of their university training. Across both states, a broad focus on cognitive, socioemotional, perceptional, and linguistic abilities as well as motivation is recommended for the diagnosis of reading and spelling difficulties (Sekretariat der Ständigen Konferenz der Kultusminister der Länder in der Bundesrepublik Deutschland, 2007).

### Materials

#### Teachers

In a personalized questionnaire, teachers indicated for each of their participating students whether a reading intervention was “not necessary,” “potentially necessary,” or “definitely necessary.” This type of rating had previously been applied in a study focusing on teachers’ ability to identify children with dyscalculia (Fischer et al., 2015). The approach mirrors the pragmatic decision teachers make about recommending students for additional reading intervention programs. Teachers were also asked how strongly they generally relied on different types of information (i.e., reading abilities, linguistic abilities, external referral) to reach their decision on a scale from 1 (*not at all*) to 5 (*absolutely*) (see Table 5 in the “Results” section). In this questionnaire, spelling was not included as an option. In the development of the questionnaire, the aim was to avoid suggestive questions and to focus on skills that could explain reading difficulties rather than skills that might be comorbid with reading difficulties. Teachers were also invited to report additional sources of information they based their decisions on. Teachers also indicated whether they used different criteria for different students.

#### Students

In group sessions, children participated in paper–pencil, standardized, normed assessments for word reading, sentence reading, text reading (Ein Leseverständnistest für Erst- bis Siebtklässler – Version II [A Reading Comprehension Test for First to Seventh Grade Students–Version–II][ELFE-II]; Lenhard et al., 2017), and spelling (Salzburger Lese und Rechtschreibtest [Salzburger Reading and Spelling Test][SLRT-II]; Moll & Landerl, 2010). In the individual sessions, all tasks were presented with HP 625 Laptops. Children were tested on standardized, normed tests of phonological awareness (Basiskompetenzen für Lese- und Rechtschreibleistung [Basic Competencies for Reading and Spelling Abilities [BAKO]; Stock et al., 2003) and vocabulary size (Wortschatz- und Wortfindungstest für 6-10-Jährige [Vocabulary and Word Retrieval Test for 6- to 10 -year-olds] [WWT]; Glück, 2011). In general, all of the standardized tests are frequently used in Germany and can be considered as reliable and valid. However, the time point of the assessment of the norm data differs slightly between tests. For example, the norm data for the reading abilities were collected at the beginning of the school year (i.e., at the same time as our data collection), while the norm data of the spelling test were collected 2 months later. This must be kept in mind when interpreting sample-specific statistics displayed in Table 2 and the overall prevalence of students with low test scores in Table 3. Reliabilities for each assessment are summarized in Table 2, and correlations are provided in Table A1 in the appendix. Below, we elaborate on test procedures. All tests also included practice trials.

**Table 2.** Descriptive Statistics and Reliability for Tasks Administered on a Student Level.

Task	*n*	*M*	*SD*	Minimum	Maximum	Reliability
Group sessions						
Word reading	697	35.68 (47.18)	10.55 (9.23)	0 (25)	67 (74)	.96^a^
Sentence reading	697	14.9 (47.6)	6.34 (10.26)	0 (25)	33 (75)	.95^a^
Text reading	697	9.06 (46.9)	5.1 (11.08)	0 (25)	26 (75)	.91^a^
Spelling	697	25.91 (40.08)	10.98 (12.06)	0 (20)	48 (66)	.94^b^
Individual sessions						
Phonological awareness	370	4.18 (35.87)	2.10 (8.98)	0 (20)	8 (58)	.67^b^
Vocabulary size	370	16.13 (31.81)	8.82 (20.16)	0 (0)	35 (65)	.90^b^

*Note.* Standardized T-values are included in parentheses.

a Odd–even split-half reliability with 1,000 simulations and adjusted by the Spearman–Brown prophecy formula. ^b^Cronbach’s alpha.

**Table 3.** Numbers of Students Selected for Different Levels of Eligibility for Reading Interventions by Teachers and Standardized Tests.

Teachers’ rating: Intervention is …	Standardized tests’ rating			
No difficulties	Mild difficulties	Severe difficulties	Sum
	Reading on the Word Level^a^			
… not necessary	322	22	8	352
… potentially necessary	137	28	7	172
… definitely necessary	98	37	38	173
Sum	557	87	53	388^e^
	Reading on the Sentence Level^b^			
… not necessary	328	18	6	352
… potentially necessary	135	25	12	172
… definitely necessary	82	42	49	173
sum	545	85	67	402^e^
	Reading on the Text Level^c^			
… not necessary	321	22	9	352
… potentially necessary	116	26	30	172
… definitely necessary	72	42	59	173
Sum	509	90	98	406^e^
	Spelling^d^			
… not necessary	288	23	41	352
… potentially necessary	72	18	82	172
… definitely necessary	32	16	125	173
Sum	392	57	248	431^e^

*Note*. Shaded cells for each subskill represent cases in which tests and teachers did not overlap in their estimation. Dark shades represent a higher achievement estimation by tests in comparison to teachers’ estimations. Light shades represent a lower achievement estimation by tests in comparison to teachers’ estimations.

a κ_w_ = 0.28. ^b^κ_w_ = 0.37. ^c^κ_w_ = 0.242. ^d^κ_w_ = 0.56. ^e^The figure on the bottom right of the respective contingency table represents the sum of the students for whom teachers and assessments reached the same conclusion. The sum of students overall was *N* = 697.

##### Word reading

In a speeded test, children were asked to select the matching word to a picture from a group of four words. Some of the distractor words were phonological or orthographic neighbors or semantically related to the target. The maximum score was 75 correctly matched words within 3 min.

##### Sentence reading

Children were asked to choose one out of five words to complete a sentence. Distractor words were of the same part of speech and often phonologically, orthographically, or semantically related. Sentences varied in complexity. Children had 3 min to answer up to 36 sentences.

##### Text reading

Children had 7 min to read up to 17 small passages with varying length and complexity. They were asked to choose the correct out of four statements about the passages. Overall, they could answer up to 26 items.

##### Spelling

Children were presented with 48 written sentences that each missed one word. Each sentence was read aloud, including the missing word. Then the missing word was repeated and children wrote the word into the blank spaces. Regular and irregular errors in grapheme–phoneme conversion and errors in capitalization were counted. In Table 2, the number of correctly spelled words is reported.

##### Phonological awareness

Children were presented with a string of four words or pseudowords and asked to identify the odd word either based on the onset (*n* = 4) or offset (*n* = 4) of the word. Sets of words were presented from prerecorded audio files. The raw score was the sum of all correctly answered items.

##### Vocabulary size

Children were asked to produce 40 nouns, verbs, or adjectives represented by pictures on a computer screen. Twelve items differed according to age (older or younger than 9 years). The raw score was the sum of all correct answers. The test provides a list of synonyms that deviate from the target but are still counted as correct.

## Results

### Agreement Between Teachers’ Ratings and Standardized Tests

In a first step, the agreement was calculated between teachers’ ratings and the estimations of standardized word reading, sentence reading, text reading, and spelling tests. To this end, three groups were formed based on children’s standardized scores in the respective assessments, which corresponded to the three steps in the teachers’ ratings. Children with *T*-values below 35 were grouped as “definitely needing” a reading intervention, children with *T*-values between 35 and 40 as “possibly needing” a reading intervention, and children with a *T*-value of over 40 as “not needing” an intervention. We chose these thresholds based on the guidelines for diagnosing reading and spelling disorders of the German Society for Child and Youth Psychiatry (Deutsche Gesellschaft für Kinder- und Jugendpsychiatrie e.V [DGKJP], 2015). These guidelines recommend thresholds of 1.5 standard deviations below age or class norms (i.e., a *T*-value of 35) for a specific literacy skill (i.e., specific reading skill, spelling) and a threshold of 1 standard deviation below age or class norms (i.e., a *T*-value of 40) if one or more other reading-related skills already indicate a deficit. While teachers themselves are unlikely to follow these specific guidelines, they are used by school psychologists, who teachers can contact for counseling and support with regard to the identification process. The DGKJP criteria further closely resemble the ones used in definitions of reading or spelling disorders in global guidelines (APA, 2013; WHO, 2020), manuals of standardized assessments that teachers have access to (e.g., ELFE-II; Lenhard et al., 2017), or scientific reports of clinical prevalence (Moll et al., 2014).

For each skill, weighted Cohen’s Kappa (κ_w_; Cohen, 1968) with disagreements weighted according to their squared distance from perfect agreement was calculated to compare teachers’ ratings with the groupings based on standardized scores. Weighted Kappa was calculated in R (R version 3.6.1; R Core Team, 2019) using the {kappa2} function from the irr package (Gamer et al., 2019). Results of these analyses are presented in Table 3.

Results in Table 3 show that, compared with standardized tests, teachers had a very high base rate of identifying children in need of a reading intervention. They identified about 24.8% of children to definitely qualify for a reading intervention and about 24.7% to possibly need a reading intervention. Thus, teachers on average classified nearly half of their students as potentially needing an intervention. It is in turn not surprising that the agreement between teachers and standardized test was highest in the “no reading difficulties” category. To interpret the average weighted agreement, we followed the guideline by Landis and Koch (1977). According to this guideline, κ < 0 indicates no agreement, κ = 0–0.20 indicates a slight agreement, κ = 0.21–0.40 indicates a fair agreement, κ = 0.41–0.60 indicates a moderate agreement, κ = 0.61–0.80 indicates a substantial agreement, and κ = 0.81–1 indicates an almost perfect agreement. Following this guideline, the agreement between teachers’ ratings and test criteria was fair for word and sentence reading abilities, and moderate for both text reading abilities and spelling.

In a next step, it was analyzed whether these differences between the different agreement scores were significant. To this end, bootstrapped distributions of κ_w_ with 1,000 random drawings were estimated for κ_w_ of each literacy skill using the {boot} function from the boot package (Canty & Ripley, 2019). Then, the bootstrapped distributions of the agreements (κ_w_) were subtracted from each other to create distributions representing the estimated differences between the respective agreements. For these distributions, 95% confidence intervals (CIs) were calculated to determine whether distributions deviated significantly from each other. As distributions with lower agreements were always subtracted from distributions with higher agreements, distributions did differ significantly if the lower bound of the 95% CI was greater than 0. Results displayed in Table 4 show that the agreement between teachers and standardized tests was significantly higher for the spelling test than for all of the reading tests. Also, agreement between teachers’ ratings and the text and sentence reading test was significantly higher than between teachers’ ratings and the word reading test.

**Table 4.** Estimated Differences of Agreements Between Teachers’ Decisions and the Respective Standardized Reading or Spelling Test.

Differences between agreements	*M*	*SD*	CI.lw	CI.up
Spelling versus word reading	0.27	0.04	0.19	0.36
Spelling versus sentence reading	0.18	0.04	0.1	0.27
Spelling versus text reading	0.14	0.04	0.05	0.22
Sentence reading versus word reading	0.09	0.04	0.01	0.18
Text reading versus word reading	0.14	0.05	0.05	0.23
Text reading versus sentence reading	0.05	0.04	−0.04	0.13

*Note.* CI.lw = lower bound of 95% confidence interval; CI.up = upper bound of 95% confidence interval.

In summary, results indicate that teachers’ ratings of children needing a reading intervention corresponded more strongly with children’s spelling abilities than with their reading abilities. Within different reading components, data indicated a gradient, suggesting that agreement was highest with text reading abilities, followed by sentence and word reading abilities, while only the difference between text and word reading and sentence and word reading reached significance.

### Indicators for Teachers’ Decision Making

We took three steps to analyze which information teachers used to guide their decisions about the children’s need of a reading intervention. Teachers’ self-reports were examined to find out the type of information teachers relied on. Multilevel models were fitted to elicit students’ literacy skills that were associated with teachers’ decisions and which showed the strongest association. Finally, multilevel models were fitted to find out whether teachers’ estimations were biased by the average level of skill in their own classroom.

#### Teachers’ self-reports

Table 5 summarizes the descriptive statistics of teachers’ self-reports regarding the type of information they relied on in their estimation of their students’ need of remedial reading intervention, rating their degree of reliance on information on a scale from 1 (*not at all*) to 5 (*absolutely*). Results show that teachers reported relying mostly on outcome data like basic and advanced reading abilities and linguistic abilities connected to reading (i.e., phonological awareness) and, to a lesser extent, on vocabulary. Second, they also relied on input data such as their impression of children’s motivation and self-concept with regard to reading and the impression they had of children’s home learning environment. On average, the type of information they relied least on was school grades and external referral. Additional sources that were reported by teachers (*n* = 4) were learning progress assessment, self-developed individualized assessments, or speech discrimination abilities. None of the teachers listed spelling as an additional source of information for their decision.

**Table 5.** Teachers’ Self-Reports Regarding Data They Relied on to Reach Decision About Student’s Need for Reading Intervention.

Type of information	*n*	*M*	*SD*	Minimum	Maximum
Basic reading abilities	53	4.64	0.74	2	5
Advanced reading abilities	53	4.34	0.85	2	5
Linguistic abilities^a^	53	4.06	1.05	1	5
Vocabulary size	53	3.08	1.05	1	5
Motivation and self-concept	53	3.21	1.01	1	5
School grade	53	2.81	1.23	1	5
External referral^b^	52	2.87	1.28	1	5
Learning environment outside of school^c^	52	3.21	1.04	1	5

a That is, phonological awareness, grammatical understanding. ^b^That is, parents, colleagues, therapists. ^c^That is, books at home, parents’ vocabulary.

#### Students’ literacy and literacy-related skills

Based on teachers’ self-reports and the analysis of the agreement between teachers’ ratings and standardized assessment, students’ reading, spelling, phonological awareness, and vocabulary assessment data were included as independent variables that might be associated with teachers’ ratings. As the data from students were nested in data from teachers, multilevel models were fitted to account for the variability in decision making between teachers (Level 2) in addition to the variability between students (Level 1). The models were fitted using the {lmer} function from the lme4 package (Bates et al., 2015). The dependent variable was teachers’ decision about each student’s need of a reading intervention, which was treated as a continuous variable from 1 to 3, with higher numbers indicating a stronger need and thus presumably lower reading and/or spelling skills. Teachers (= classes) were added as random effect. Models were fitted in a hierarchical manner, separately for the full sample (group sessions) and for the smaller sample (additional individual sessions). Standardized values of word reading for each student (i.e., basic reading abilities) were added as the first fixed effect, followed by advanced reading abilities, spelling, phonological awareness abilities, and vocabulary (see Table 6).

**Table 6.** Results of Hierarchical Multilevel Regression Analyses Predicting Teachers’ Decision.

Model	Step	Predictor	Group session sample				Individual session sample			
			β	*SE*	*t*	Pseudo-*R*^2^	β	*SE*	*t*	Pseudo-*R*^2^
1	1	Word reading	−.04	.003	−16.27***	.30***	−.04	.005	−8.17***	.17***
2	1	Word reading	−.02	.003	−5.56***	.14***	−.02	.006	−4.01***	.09***
	2	Text reading	−.03	.003	−9.88***		−.03	.005	−5.52***	
3	1	Word reading	−.01	.004	−1.82	.14***	−.01	.006	−1.15	.12***
	2	Text reading	−.02	.003	−6.25***		−.02	.005	−4.24***	
	3	Spelling	−.03	.003	−9.87***		−.03	.005	−6.43***	
4	1	Word reading					−.01	.006	−0.97	.06***
	2	Text reading					−.02	.005	−3.94***	
	3	Spelling					−.02	.004	−5.27***	
	4	Phonological awareness					−.02	.004	−4.59***	
5	1	Word reading					−.01	.006	−1.21	.04***
	2	Text reading					−.01	.004	−3.29**	
	3	Spelling					−.02	.004	−5.39***	
	4	Phonological awareness					−.02	.004	−4.10***	
	5	Vocabulary					−.01	.002	−3.68***	

*Note*. * *p* < .05. **p < .01. ***p < .001.

A variety of measures were calculated to assure the validity of the models and extract the explained variance. For each model, a factor for variance inflation using the {vif} function from the car package (Fox & Weisberg, 2019) was calculated to check for multicollinearity. Results of the test for sentence reading were strongly correlated with word and text reading (see Table A1). This led to variance inflation of the sentence reading effect so only reading on the text level was included in the final models as a measure of advanced reading abilities. In the final models, there was no indication for variance inflation with all variance inflation factors being smaller than 2 and correlations between predictors being low to medium (see Table A1).

Furthermore, pseudo-*R*^2^ for Level 1 effects was calculated using the {r.squaredLR} function from the MuMin package (Bartoń, 2019) and is reported for each model to indicate the mean percentage of additionally explained Level 1 variance across all predictors in comparison to the prior model, when an additional predictor was added. For the first model, the previous model was the null model (see Table 6). Finally, model fit comparisons were calculated with the {anova} function from the stats package (R Core Team, 2019) after each additional step to compare the fit of the new model with the previous model. Significant results are indicated by asterisks in the pseudo-*R*^2^ column and reported in detail in Table A2 of the appendix.

In line with the results of the analysis of agreement between teachers and standardized assessments, the results of the hierarchical multilevel analysis showed that children’s spelling abilities explained additional variance in teachers’ ratings over and above their reading skills. Notably, when both text reading and spelling abilities were included in the model, word reading skills ceased to be a significant predictor of teachers’ ratings. Among reading skills, the analysis indicates that a larger amount of variance was explained by advanced reading abilities (e. g., text reading) in comparison with basic reading skills (e.g., word reading). Furthermore, additional variance was explained by reading-related abilities such as phonological awareness abilities and vocabulary size.

#### Effects of average class level

As in the previous analyses, teachers’ decisions about their student’s need of reading intervention were the dependent variable and teachers (= classes) were added as random effects. However, in addition to the standardized mean for each student, the class means of each predictor were entered as an additional fixed effect. It was thus possible to test whether the average skill level in each class had an impact on teachers’ ratings. These analyses were performed for the group session sample only, given that the reference group for the teachers was the full class.

Results indicated effects of average class levels for all predictors (see Table 7). As expected, differences in the directions of effects were found between student-level and class-level effects. Student-level results indicated that across all classes, children with better standardized text reading scores were less likely to be identified as needing a reading intervention. Class-level results indicated that equally able students were more likely to be selected as needing a reading intervention when the average ability level of other students in their class was high than when the average ability level of other students in their class was low. Thus, results indicate that teachers consider the average ability level of the children in their classroom when deciding which of their students need an additional reading intervention.

**Table 7.** Average Class-Level Effects in Addition to Student-Level Effects on Teachers’ Ratings of the Need for Reading Interventions of Their Students.

Predictor	Student-level effect			Class-level effect		
	β	*SE*	*t*	β	*SE*	*t*
Word reading	−.05	.003	−16.04***	.02	.008	2.35*
Text reading	−.05	.003	−18.61***	.02	.006	3.18**
Spelling	−.05	.002	−21.46***	.03	.006	4.88***

*Note.* *p < .05. **p < .01. ***p < .001.

#### Summary

Results indicate that students’ spelling abilities, along with phonological awareness and vocabulary, explained variance in teachers’ ratings over and above students’ reading skills. Finally, class-level effects indicate that teachers’ decisions were influenced by the average ability level of the children in their classroom when deciding which of their students need an additional reading intervention.

## Discussion

The aim of this study was to explore how teachers who do not need to follow a formalized procedure decide on their students’ need of a reading intervention. To this end, we evaluated how far teachers’ decisions overlapped with indicators of standardized assessment of reading and reading-related skills, which skills are associated with teachers’ decisions, which type of data teachers relied on most, and whether teachers’ decisions were biased toward proximal cues such as the average achievement level of the classroom. The study expanded on previous research in several ways. First, we focused on teachers’ unguided decisions in an educational environment in which the diagnostic process of identifying children in need of a reading intervention is not formalized. Second, we compared teachers’ decisions with a number of standardized tests that each provide an objective indication of difficulties in reading and reading-related skills as opposed to focusing on just one skill. Third, we included data about the type of information teachers reported to use and explored decision biases.

### Overlap of Teachers’ Decisions With Standardized Clinical Criteria and Identification Rate

In line with previous literature (e.g., Martin & Shapiro, 2011), we found an at best moderate agreement between teachers’ decisions and categories based on the standardized assessment of reading or spelling skills. Moreover, also in line with previous literature (Begeny et al., 2008), the agreement with standardized assessments was relatively high in identifying students who did not need a reading intervention. In comparison with standardized assessment, teachers identified a large proportion of students (almost 50%) as being “definitely” or “possibly” in need of a reading intervention. Nevertheless, between 25% and 40% of these children achieved a *T*-value of above 40 in standardized tests of reading or spelling. In contrast to previous reports (e.g., Martin & Shapiro, 2011), our findings thus suggest an underestimation of students’ achievement by teachers rather than an overestimation. Clinical prevalence rates in our study were slightly higher than prevalence rates in previous German samples (e. g., Moll et al., 2014). Thus, the standardized criteria already accounted for an increased prevalence of reading difficulties, but teachers’ percentage of identification was even higher.

One factor that might have contributed to teachers’ high identification rate could be that they took into account results of Germany-wide standardized assessments that use alternative approaches to clinical criteria and have had a large media presence (Internationale Grundschul-Lese-Untersuchung [IGLU]/Progress in International Reading Literacy Study [PIRLS], Hußmann et al., 2017; Institut zur Qualitätssicherung im Bildungswesen [Institute for Educational Quality Improvement] [IQB] Bildungstrend [Educational Trend], Stanat et al., 2017). In these assessments, five thresholds of competency levels are applied (Pant et al., 2010). Standardized assessment scores below the second competency level are interpreted as a competency below the minimal standard and scores below the third competency level are interpreted as a competency below the regular standard. These competency thresholds include a larger percentage of students than clinical criteria. For example, a competency below Level 2 in the reading assessment of 2016 (Stanat et al., 2017) roughly translates to 14% of students (score of 390 and *T*-value of 39) and a competency level below 3 roughly translates to 36% of students (score of 464 and *T*-value of 46.4). However, even compared with these more liberal thresholds of difficulties, teachers still underestimated their students’ reading performance. We speculate that teachers’ estimations could be explained by additive estimations of observations of reading, spelling, and possibly also language difficulties. Thus, teachers might have deemed students with problems in either of these domains as qualifying for a reading intervention.

### Types of Data Teachers Might Use for Their Decision

Self-reports indicate that teachers relied most strongly on outcome data followed by input data. Teachers rarely referred to school grades or external referral (i.e., by parents or therapists). Among outcome data, results of the multilevel models showed that variance of teachers’ decisions was explained by spelling skills over and above reading abilities. An additional amount of variance was explained by phonological awareness and vocabulary size. Among reading abilities, advanced reading abilities (i.e., text reading) explained more variance than basic reading skills (i.e., word reading). Based on these findings, we conclude that with regard to abilities that are reported in scientific literature (e.g., Castles et al., 2018; Snowling & Hulme, 2012), clinical standards (e.g., APA, 2013; WHO, 2020), or regional guidelines (HKM, 2017a), teachers relied on a set of skills that is commonly associated with reading disorders. Surprisingly, the skill teachers’ decisions about which student needed a *reading* intervention most distinctly related to, was children’s spelling abilities. Yet, previous studies, general clinical guidelines, and local guidelines have indicated that spelling problems often appear separately from reading problems. Finally, teachers’ decisions were affected by the average reading level in their classroom, which means that equally able students had different likelihoods of being selected for a reading intervention depending on the average ability level of their peers.

### Biases in Instructional Data Use

Results across all analyses indicate that teachers’ decisions about whether a child needs a reading intervention were better predicted by children’s spelling performance than by their actual reading performance. Furthermore, teachers’ decisions were related to the average achievement level of their classroom. While this result may seem surprising at a first glance, we interpreted this as a reliance on proximal cues obtained in daily practice (Brunswik, 1952; Dhami et al., 2004; Gelderblom et al., 2016). Errors in spelling are much more perceivable for teachers in daily practice than errors in spoken language or even silent reading. In addition, data on students’ spelling skills are regularly obtained by teachers through common practices such as graded dictations. Presumably, teachers generalize children’s spelling problems in terms of poor overall literacy or language abilities.

This conclusion is further supported by significant class-level effects, which indicate that children with the same test scores were more likely to be identified as “needing reading intervention” when the average ability level of other students in their class was high in comparison with classes in which the average ability was low. Furthermore, within reading abilities, teachers’ decisions were explained by advanced over and above basic reading abilities, which are taught as part of the third-grade curriculum in Germany. In summary, results point to cognitive biases explained by a reliance on proximal cues obtained in daily practice. These cues might skew teachers’ decisions and lead to misidentifications of children with regard to reading intervention.

### Class-Level Effects

Results show significant class-level effects on each of the skills that were assessed in the large sample. These findings suggest that, to reach a decision about an individual child, teachers considered the average level of their class in a particular skill. While this outcome might not be surprising from a practical point of view, it is clearly at odds with an objective, clinical diagnostic process. A significant class-level effect implies that children in a class with a high average reading level were more likely to be identified as being in need for reading intervention, whereas the opposite is true for children in a class with a low average reading level. This finding provides additional evidence for our interpretation that teachers relied on proximal cues obtained in daily practice.

### Results in Relation to Definitions of Reading Disorders

Teachers’ reliance on reading as well as on spelling and more general language abilities, suggests that teachers, on average, did not distinguish between reading and spelling disorders. Such a distinction can, however, be found in local guidelines for teachers (HKM, 2017a) as well as—to some extent—in the *DSM-5* (APA, 2013). In line with these standards, results of standardized assessments indicate isolated reading and spelling disorders in student samples. For example, the number of children with identified spelling difficulties exceeded the number of children with reading difficulties (see Table 3). This suggested the presence of cases of comorbid reading and spelling disorders as well as of isolated spelling disorders (Moll et al., 2014; Moll & Landerl, 2009; Snowling & Hulme, 2012).

Similarly, cases of dyslexia and poor comprehension also seemed to be present, because more students were identified with severe reading difficulties on the text than on the word level (see Table 3). This indicates an additive effect of dyslexia and poor comprehension, which aggravates difficulties on reading comprehension tasks. Teachers decisions were also predicted by phonological awareness and vocabulary—the former being associated with dyslexia or spelling difficulties, while the latter being related to poor comprehension (Gough & Tunmer, 1986; McBride, 2019; Moll et al., 2014; Snowling & Hulme, 2012). Results, therefore, indicate that teachers on average searched for a general indication of reading or spelling difficulties and possibly general language difficulties instead of specifically defined cases of reading difficulties. To conclude, with regard to specific definitions of reading disorders, teachers’ decision-making process may lead to an unspecific mapping of children onto reading interventions (Reynolds & Shaywitz, 2009; Snowling, 2013).

### Future Directions in Research and Practical Implications

This study was an exploratory approach to attempt resolving the question which data or information teachers use, to reach a decision about their student’s need for a reading intervention. This question is highly relevant for teachers’ own orientation toward criteria that might help them to reach their decision about reading intervention, especially if there is no formalization of the identification process. Likewise, the study conveys important information for school psychologists or specialized educators who are likely to adhere to clinical criteria and can profit from information that will enable them to communicate better with teachers that seek their advice. An answer to this question is also important for stakeholders and educational researchers, who within their capacities need to be sensitive to different perspectives within the educational system and reach compromise. Finally and importantly, this type of research is also relevant for parents and children who are likely most affected by the way a decision about the need for a reading intervention is made. Thus, it is important to point out open questions that were not answered by this study but could be addressed by future research.

Results of this study indicate that teachers’ decision-making processes might have been more complex and individualized than we could elucidate. For example, in their self-reports, teachers reported that they also relied on students’ motivation for reading and their self-concept about their reading abilities. Thus, future studies could benefit from including more indicators from different types of data (Lai & Schildkamp, 2013). These data could further elucidate between-student variability with regard to teachers’ self-reported information. For example, self-reports about which data teachers relied on could be collected for each student. Furthermore, studies could include more detailed questions about what kind of criteria teachers rely on in their identification process. Self-reports could assess whether teachers rely on clinical or competency-level criteria, if they have developed their own set of criteria and what skills they include in their observations besides reading (i.e., spelling, language abilities, behavioral observations). Finally, structural information about reading interventions could be collected to explore how teachers’ decisions map onto abilities that are trained in the respective reading intervention.

Our findings indicate that a significant proportion of children identified as “definitely needing” a reading intervention might be selected for an intervention that does not really fit their needs. This could be either children who have a specific learning disorder but share an intervention program with children who have different or no issues, or children who do not have a disorder at all but are submitted to an intervention program together with children with specific needs. In both cases, the intervention could have negative effects on children’s reading or spelling development (e.g., being ineffective or demotivating). It is important that future studies also focus on how children with or without learning difficulties are affected by mismatched interventions, and which children are most likely to be misidentified.

This study offers several indicators with regard to how teachers’ decision-making processes could be supported. Regarding our findings that teachers rely on proximal cues, guided questionnaires that ask teachers whether their students have trouble with reading or spelling (or both) could help identify necessary additional information. Giving teachers scenarios (i.e., “Does your student have trouble sounding out fantasy words?”; “How does the student’s reading level compare to students in other classes?”) could guide teachers in their decision-making process. Studies with guided questionnaires have shown that teachers can reliably identify at-risk students (Connor et al., 2013; Titley et al., 2014). Teachers’ prior knowledge can influence the diagnostic and intervention process, too (e.g., Piasta et al., 2009). Therefore, the guided approach could be combined with professional development courses informing teachers about reading and spelling disorders and possible biases they need to look out for in their decision-making process as well as support they can seek out (specialized educators, school psychologists, therapists).

Teacher training on the identification of children with a need for additional reading training could likewise be intensified. In Germany, teacher training is split up between university studies and an 18-month practical traineeship after university training. While university programs typically teach at least some knowledge of diagnostics for children at risk of developing learning disabilities, practical traineeship mainly focuses on curriculum-specific didactics. It is clear that preservice teachers should be informed about how learning difficulties are diagnosed as well as about effective intervention programs for *specific* learning difficulties (see, for example, Ise et al., 2012). University training could include courses that provide knowledge about current definitions of specific learning disorders, different diagnostic criteria, and common diagnostic biases. Practical traineeship could include observing diagnostic sessions of specialized educators, school psychologists, or therapists.

### Limitations

This study had several limitations. First of all, the study was clearly exploratory in nature. As a result, in retrospect, several additional information should have been collected. For example, spelling was not included as a fixed source of information in teachers’ self-report. Given that our results strongly suggest that the students’ spelling ability is an important source of information for teachers’ decisions, future studies should collect data on teachers’ self-rated reliance on spelling skills for diagnosing reading difficulties. Moreover, we asked teachers to indicate whether or not children needed a reading intervention without any further specification. Future studies could ask them directly to identify children with specific reading comprehension or spelling difficulties and compare those ratings with respective standardized assessments. Finally, this study has some limitations with regard to the comparison of teacher decisions and criteria used on clinical assessments. The direct comparisons of teachers’ decision and clinical criteria reflect the comparison of estimations applied in school versus estimations that would be considered in a clinical assessment (e.g., by a school psychologist or therapist). In an unformalized environment, it is very difficult to find criteria that will apply to all teachers. Therefore, the results of this first exploratory result need to be interpreted carefully, and future studies should try to find more individualized approaches or combine qualitative and quantitative approaches to identify information and criteria that teachers rely on.

### Summary

We compared teachers’ decisions about their students’ need for a reading interventions with clinical classifications of standardized reading and spelling tests, and explored how teachers reached their decisions. We found that teachers’ decisions overlapped most strongly with classifications by standardized spelling assessments as opposed to reading assessments. Using a hierarchical linear model, we found that students’ spelling abilities, along with phonological awareness and vocabulary, explained variance in teachers’ ratings over and above students’ reading skills. In addition, teachers’ decisions were oriented toward the average achievement level of their class. Research on human decision making might serve to explain such findings, which suggests that even professionals rely on proximal indicators (here: spelling performance, advanced reading training, and classroom environment) and misinterpretations are possible regarding the predictive power of these indicators for related skills. Furthermore, teachers on average presumably relied on a global indication of both reading and spelling difficulties instead of specific indicators of reading difficulties. These findings have practical relevance: They suggest that teachers’ decision making might be linked to ineffective mapping of children to interventions. By consequence, children might be assigned to interventions that are unspecific with regard to the underlying causes of their reading difficulties—or children might receive a reading intervention who would actually need a spelling intervention. Furthermore, initial hints can be gleaned for designing effective diagnostic teacher training programs, which should consider common biases as well as biases that are specific to diagnosing isolated difficulties in reading or writing.

## Appendix

**Table A1.** Correlations of Teachers’ Ratings and (Standardized) Predictors.

Variables	1.	2.	3.	4.	5.	6.	7.	8.	9.	10.
1. Teachers’ rating	1									
2. Rating word reading	.36	1								
3. Rating text reading	.46	.53	1							
4. Rating spelling	.56	.43	.47	1						
5. Word reading	−.51	−.68	−.51	−.58	1					
6. Sentence reading	−.6	−.61	−.63	−.65	.85	1				
7. Text reading	−.57	−.49	−.77	−.56	.71	.83	1			
8. Spelling	−.59	−.48	−.51	−.85	.69	.75	.69	1		
9. Phonological awareness	−.39	−.28	−.28	−.35	.3	.37	.31	.4	1	
10. Vocabulary size	−.2	−.09^c^	−.16^a^	−.1^c^	.07^c^	.22	.25	.12^b^	.18	1

*Note.* Most correlations *p* < .001.

a *p* < .01. ^b^*p* < .05. ^c^*p* > .05; see Table 2 for respective *n*.

**Table A2.** Results of Model Comparisons of Stepwise Regression.

Model comparison	χ^2^	*df*	*p*
Group session sample			
1 versus 2	90.77	1	<.001
2 versus 3	88.01	1	<.001
Individual session sample			
1 versus 2	28.94	1	<.001
2 versus 3	38.07	1	<.001
3 versus 4	20.44	1	<.001
4 versus 5	13.01	1	<.001
